# Optimization of an Active Edible Coating Based on Cassava Starch (*Manihot esculenta* Crantz) and Lemon Verbena Essential Oil (*Aloysia citrodora*) for the Sustainable Extension of the Shelf Life of Cape Gooseberries (*Physalis peruviana* L.)

**DOI:** 10.3390/foods15091459

**Published:** 2026-04-22

**Authors:** Orlando Meneses Quelal, Yamileth Pozo Orbe

**Affiliations:** Carrera de Alimentos, Universidad Politécnica Estatal del Carchi, Tulcán 040101, Ecuador; yamileth.pozo@upec.edu.ec

**Keywords:** *Physalis peruviana* L., active edible coating, cassava starch, lemon verbena essential oil, preservation, optimization

## Abstract

This study addresses the imperative need to extend the shelf life of the cape gooseberry (*Physalis peruviana* L.), a highly perishable yet nutritionally valuable fruit, through the development and optimization of active edible coatings (ECs). The synergy between cassava starch (*Manihot esculenta* Crantz) and lemon verbena essential oil (*Aloysia citrodora*), both bioactive components, was investigated for the formulation of protective coatings. A 2^2^ factorial design explored the impact of cassava starch concentrations (8% and 10% *w*/*v*) and lemon verbena essential oil (LVEO) (1% and 3% *v*/*v*) on the sensory acceptability of coated cape gooseberries. Through binomial logistic regression analysis, it was determined that the formulation with 10% cassava starch and 3% LVEO (T4) exhibited significantly superior sensory acceptability, optimizing the perception of color, odor, flavor, texture, and overall appearance. This optimized formulation (T4) demonstrated a significant improvement in extending the shelf life of cape gooseberries up to 27 days at 10 °C, which is comparable to or exceeds values reported in previous studies on starch–based coatings in similar fruits (e.g., 15–21 days depending on formulation and storage conditions). This performance also exceeded the storage periods observed at 6 °C (6 days) and 8 °C (20 days). Physicochemical analyses revealed remarkable stability of pH and titratable acidity, as well as effective control of moisture loss and the maturity index, even at higher temperatures. Crucially, T4 exhibited superior antimicrobial activity, with a significant reduction in molds, yeasts, and total aerobes, particularly at 10 °C, suggesting an optimal synergistic interaction between the coating and the LVEO under slightly warmer storage conditions. These findings contribute to the advancement of sustainable preservation strategies of cape gooseberries, offering a sustainable solution that reconciles efficient shelf-life extension with consumer acceptability and optimizes storage conditions, with significant implications for reducing food waste and enhancing the global marketability of this fruit.

## 1. Introduction

The growing global demand for fresh and minimally processed foods, combined with the imperative need to mitigate food waste, has driven intense research into post-harvest technologies. Among emerging strategies, edible coatings (ECs) have positioned themselves as a sustainable and effective alternative for extending the shelf life of perishable fruits and vegetables [1]. These externally applied biopolymers act as semi-permeable barriers, controlling respiration, reducing transpiration, and minimizing microbial growth—key factors in the spoilage of fresh produce [2]. Their biodegradable nature and the possibility of incorporating bioactive compounds make them particularly attractive to the food industry, aligning with consumer trends toward more natural and safer products [3].

The cape gooseberry (*Physalis peruviana* L.), also known as aguaymanto or goldenberry, is a tropical fruit highly valued for its nutritional profile, which includes high levels of vitamins A and C, antioxidants, and bioactive compounds with anti-inflammatory and anti-diabetic properties [4]. However, its high perishability post-harvest conditions, characterized by rapid loss of firmness, dehydration, and susceptibility to fungal attack, severely limit its marketing and distribution, generating considerable economic losses [5]. The delicate structure of its pericarp and its high metabolic rate make the cape gooseberry a particular challenge for preservation technologies. In this context, the development of specific and optimized conservation treatments for this fruit represents a critical need to unlock its potential in international markets.

Polysaccharides, such as starch, are widely studied materials for the formulation of edible coatings (ECs) due to their low cost, abundance, non-toxicity, and excellent film-forming capacity [6]. Cassava starch (*Manihot esculenta* Crantz), in particular, has proven to be a versatile polymer, capable of generating thermoplastic matrices with adjustable mechanical and barrier properties through the addition of plasticizers such as glycerol [7]. However, the effectiveness of starch-based coatings can be significantly improved by incorporating antimicrobial and antioxidant agents. Essential oils (EOs) emerge as promising candidates in this regard, given their recognized biological activity against a wide range of fungal and bacterial pathogens responsible for postharvest spoilage [8]. Lemon verbena (*Aloysia citrodora*), with its distinctive aromatic profile and potent antimicrobial properties, is presented as an essential oil of interest for applications in ECs [9].

The incorporation of essential oils into starch polymer matrices is not without its challenges. The hydrophobic nature of most essential oils can lead to compatibility issues with the hydrophilic starch matrix, affecting film homogeneity and, consequently, its mechanical and barrier properties [10]. High concentrations of essential oils can compromise the coating’s structural integrity, increasing its water vapor permeability and decreasing its tensile strength [11]. Therefore, optimizing the essential oil concentration is a critical step in achieving a balance between antimicrobial functionality and the coating’s physical integrity [12]. Previous studies have indicated that an optimal range for incorporating essential oils into starch matrices is generally between 0.5% and 2% [13], where antimicrobial activity is maximized without significant detriment to mechanical and barrier properties.

In addition to functional properties, consumer sensory acceptability is a determining factor for the commercial success of any food preservation technology [14]. Refined coatings (ECs) must be odorless, tasteless, or impart desirable organoleptic characteristics without negatively altering the perceived quality of the fresh product. The interaction between coating components, such as starch and essential oil, can influence the release of volatile compounds and, consequently, the sensory profile of the fruit [6]. Rigorous sensory evaluation, complemented by advanced statistical tools such as binomial logistic modeling, is essential to identify formulations that are not only technically effective but also highly accepted by consumers [15].

Storage temperature is another critical factor that interacts with the effectiveness of the coating. Cold chain conditions are fundamental for preserving the cape gooseberry [16]. However, optimizing temperature in the presence of an active coating can reveal unexpected synergies, such as modulating the volatility of essential oil compounds or altering the fruit’s metabolic rate. This could lead to extended shelf life even at temperatures that, without the coating, would be suboptimal [17]. Elucidating these interactions is vital for establishing efficient and sustainable storage protocols.

This study aims to develop and characterize an edible coating based on cassava starch and lemon verbena essential oil (LVEO), seeking to optimize its formulation to extend the shelf life of cape gooseberries. Through a multifactorial approach, the balance between functional properties (barrier, antimicrobial) and sensory acceptability will be explored. Finally, the efficacy of the optimized coating will be evaluated under different storage temperature conditions, with the objective of identifying the most suitable combination for post-harvest preservation of cape gooseberries, thus laying the groundwork for practical applications in the food industry.

## 2. Materials and Methods

### 2.1. Raw Materials and Fruit Conditioning

**Figure 1:**
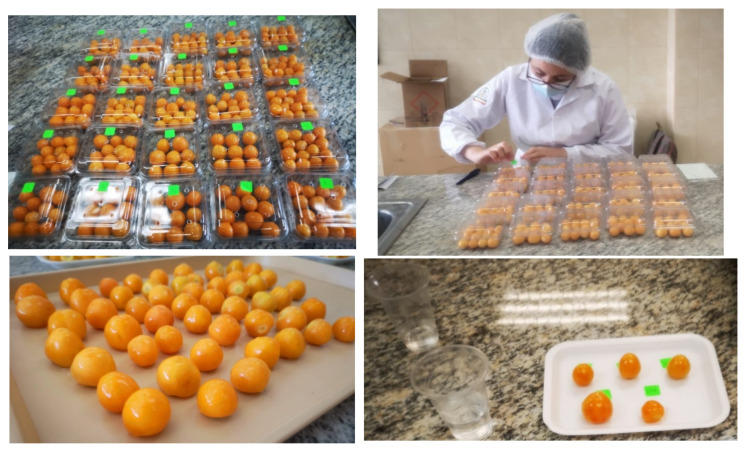
Cape gooseberries (*Physalis peruviana* L.) in a state of physiological maturity, prepared for the application of coatings.

Cape gooseberries (*Physalis peruviana* L.) at physiological maturity, characterized by a completely yellow calyx and an average diameter of 2.5 cm, were selected from local markets in Tulcán, Carchi, Ecuador. The specific cultivar was not identified. Fruits with imperfections or surface damage were discarded. To ensure food safety, the fruits were thoroughly washed with potable water and disinfected by immersion in a sodium hypochlorite solution (200 ppm) for 15 min, following standardized protocols [18]. Subsequently, the cape gooseberries were dried using forced air at room temperature and stored at 10 °C until use. Commercial cassava starch (*Manihot esculenta* Crantz), lemon verbena essential oil (*Aloysia citrodora*), food-grade, obtained from a local specialized supplier (Tulcán, Ecuador). According to the supplier’s specifications, the oil contains major components such as citral (neral and geranial), limonene, and β-caryophyllene (Figure 1).

### 2.2. Experimental Design and Preparation of Edible Coatings (ECs)

The experimental design was structured as a full factorial design (2^2^), evaluating the influence of two main factors: cassava starch concentration (8% and 10% *w*/*v*) and LVEO concentration (1% and 3% *v*/*v*). This factorial configuration generated four active coating formulations, designated T1 to T4. Additionally, a control group (T5) consisting of uncoated cape gooseberries was included to establish a baseline for comparison.

The selected concentration ranges for both factors were established based on technological functionality and previous scientific evidence. Cassava starch concentrations of 8% and 10% (*w*/*v*) were chosen because lower concentrations (<8%) tend to produce films with insufficient mechanical strength and high-water vapor permeability, whereas higher concentrations (>10%) may result in excessive viscosity, limiting uniform application and film formation.

For lemon verbena essential oil (LVEO), concentrations of 1% and 3% (*v*/*v*) were selected to represent a commonly reported effective antimicrobial level (around 1%) and an upper threshold (3%) to evaluate potential improvements in bioactivity, despite the structural destabilization often reported at concentrations above 2%.

The use of a 2^2^ factorial design allowed for an efficient evaluation of the main effects and interaction between both factors while minimizing the number of experimental runs. Intermediate concentrations were not included at this stage, as the objective was to identify an optimal region within a defined experimental domain. Further optimization could be addressed in future studies using response surface methodology.

The detailed composition of each treatment (T1–T5) is presented in Table 1. Each treatment was replicated three times, with 60 fruits per replicate, resulting in a total of 180 fruits per treatment and 900 fruits in the overall experiment, ensuring homogeneity in the initial characteristics of the raw material.

**Figure 2:**
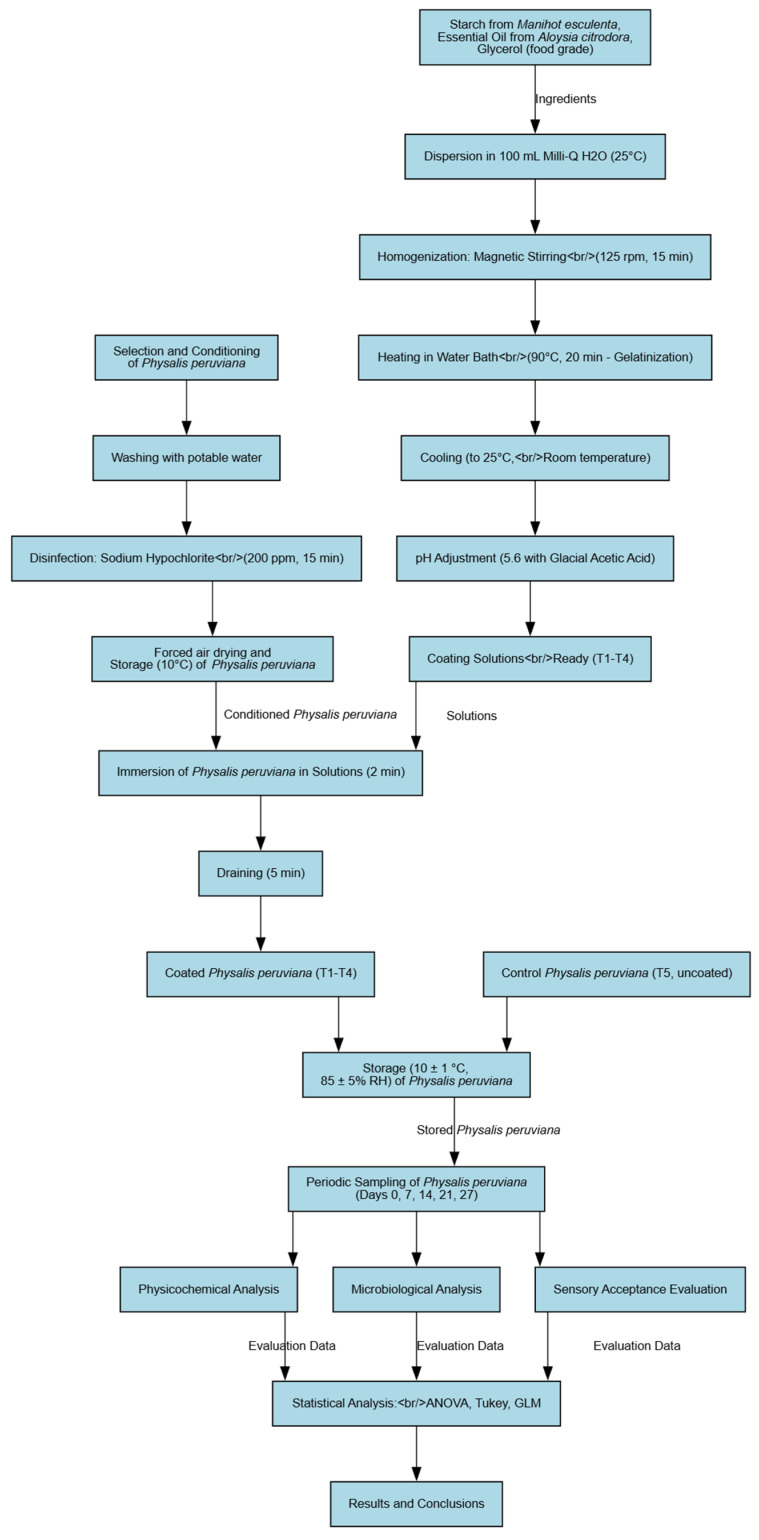
Schematic flow diagram of the process of preparation and application of ECs in cape gooseberries (*Physalis peruviana* L.) and their evaluation.

The development of the coating solutions was based on the methodology of Liu [19] and Iamareerat et al. [20] with slight modifications. Specific concentrations of cassava starch and essential oil were dispersed in 100 mL of Milli-Q distilled water (MilliporeSigma, Burlington, MA, USA) at 25 °C, along with glycerol (0.3% *v*/*v*) as a plasticizer. The mixture was homogenized at 125 rpm for 15 min by magnetic stirring using a magnetic stirrer (Model C-MAG HS 7, IKA-Werke GmbH & Co. KG, Staufen, Germany). Subsequently, the dispersion was heated in a water bath with constant stirring to 90 °C for 20 min to ensure complete starch gelatinization, which is critical for the formation of a uniform and stable polymer matrix. After gelatinization, the solution was cooled to approximately 40–45 °C, and the lemon verbena essential oil (*Aloysia citrodora*, LVEO) was incorporated at concentrations of 1% and 3% (*v*/*v*) under continuous stirring to minimize volatilization losses and ensure proper dispersion. The solution was then further cooled to room temperature (25 °C). Finally, the pH of each formulation was adjusted to 5.6 using glacial acetic acid (99.8% purity, Merck, Darmstadt, Germany), monitored with a digital pH meter, optimizing both emulsion stability and the antimicrobial activity of the essential oil. A schematic representation of the experimental design and the main methodological steps is illustrated in Figure 2.

### 2.3. Application of Coatings and Storage Conditions

The cape gooseberries, previously selected and conditioned, were carefully immersed in each of the corresponding coating solutions (T1–T4) for 2 min. This immersion time was standardized to ensure complete and uniform coverage of the fruit surface, minimizing the risk of uncoated areas. After immersion, the fruits were removed and placed on a stainless-steel mesh to allow for 5 min of drainage, removing excess coating. The fruits were then arranged on perforated polypropylene trays. The control group (T5) consisted solely of uncoated fruits, following the same handling and storage procedures to ensure comparability.

All fruits, both coated and in the control group, were stored at three controlled temperatures (6, 8, and 10 ± 1 °C) and relative humidity (85 ± 5% RH) inside a climate chamber (ICH 110 model, Memmert, Schwabach, Germany). Each temperature condition was evaluated independently to assess its effect on shelf life. These conditions simulated those of a commercial cold chain and were maintained and continuously monitored. Quality monitoring was performed at regular 7-day intervals over a total storage period of up to 27 days (days 0, 7, 14, 21, and 27), collecting random samples for physicochemical and microbiological analyses.

### 2.4. Sensory Analysis

To assess consumer acceptability, an affective sensory study was conducted with a panel of 60 untrained panelists (recruited from the student population of the State Polytechnic University of Carchi, UPEC), randomly selected and without allergies to the products. The evaluation took place in individual booths with standardized lighting in the UPEC sensory analysis laboratory. Each panelist was presented with randomly coded samples of the different treatments (coated cape gooseberries and a control). The evaluation was performed using a structured 5-point hedonic scale, where 1 represented “I dislike it very much,” 2 “I dislike it,” 3 “I neither like nor dislike it,” 4 “I like it,” and 5 “I like it very much.” The attributes evaluated individually included appearance (brightness, color), aroma (intensity, pleasantness), flavor (sweetness, acidity, off notes), and texture (firmness, juiciness), in addition to overall acceptance. Water was provided between each sample to cleanse the palate. This approach allows for a direct and representative assessment of the average consumer’s perception of the treatments.

The sensory evaluation involved voluntary participation of adult panelists and was conducted under minimal-risk conditions. All participants were informed about the objective of the study and provided their informed consent prior to participation.

### 2.5. Physicochemical Analyses

The physicochemical parameters were determined in the experimental treatments at defined intervals (days 0, 7, 14, 21, and 27) throughout storage, to quantify the effect of the coatings on the post-harvest quality of the cape gooseberry and its shelf life. All measurements were performed in triplicate for each sample.

#### 2.5.1. pH and Titratable Acidity

The pH was measured directly in a homogenized mixture of 10 g of cape gooseberry pulp (obtained from 3 fruits per replicate) with 100 mL of distilled water, using a previously calibrated digital pH meter (Hanna Instruments HI 2211, Woonsocket, RI, USA). Calibration was performed with pH 4.01 and 7.00 buffer solutions at 25 °C. This procedure was carried out according to Ecuadorian standard NTE 0389 [21]. Titratable acidity was determined by potentiometric titration of 10 mL of the homogenate with 0.1 N sodium hydroxide (NaOH) to an endpoint of pH 8.1. The results were expressed as a percentage of anhydrous citric acid, citric acid being the predominant acid in cape gooseberries, according to Ecuadorian standard NTE INEN 2485 [22]. These measurements are critical for monitoring the degree of maturity, respiration, and stability of the fruit during storage.

#### 2.5.2. Total Soluble Solids (°Brix)

The concentration of total soluble solids (TSS) was quantified using a portable digital refractometer (Atago PAL-1, Saitama, Japan), previously calibrated with distilled water at 20 °C. Four drops of macerated and filtered cape gooseberry pulp (obtained from 3 fruits per replicate) were placed on the refractometer prism to obtain a direct reading in °Brix. The results were expressed as °Brix at 20 °C, according to the Ecuadorian standard NTE INEN 0380 [23]. TSS is an important indicator of fruit maturity and sugar content.

#### 2.5.3. Moisture Content and Maturity Index

Moisture content was assessed using the gravimetric oven-drying method. Approximately 5 g of cape gooseberry pulp sample was weighed into tared aluminum capsules and dried in a circulating oven (ULM 500, Memmert GmbH + Co. KG, Schwabach, Germany) at 105 ± 1 °C until constant weight was reached (approximately 5 h). Moisture content was calculated as weight loss and expressed as a percentage (%). This analysis was performed according to Ecuadorian standard NTE INEN 265 [24]. Determinations were carried out on days 0, 7, 14, 21, and 27 for each treatment, providing a dynamic understanding of water loss and fruit dehydration.

The maturity index (MI) was calculated as the ratio between total soluble solids (°Brix) and titratable acidity (expressed as a percentage of citric acid), using the formula MI = °Brix/% Acidity, according to ICONTEC standard 4580 [25]. This index is a key descriptor of the ripening state and organoleptic quality of the fruit.

### 2.6. Scanning Electron Microscopy (SEM) Analysis

The microstructural characterization of the edible coating corresponding to the optimal treatment (T4) was performed using scanning electron microscopy (SEM). Film samples were obtained by drying the coating-forming solution under controlled conditions and subsequently cutting them into small sections (approximately 5 × 5 mm).

Prior to observation, the samples were mounted on aluminum stubs using double-sided conductive carbon tape and sputter-coated with a thin layer of gold to improve electrical conductivity.

SEM analysis was carried out using a scanning electron microscope (JEOL JSM-IT100, Tokyo, Japan) operating at an accelerating voltage of 10–15 kV. Micrographs were obtained at different magnifications to evaluate surface morphology and internal structure.

The average pore size was estimated from SEM images using ImageJ software (version 1.53, National Institutes of Health, Bethesda, MD, USA). At least three independent samples were analyzed, and multiple measurements per sample were performed to ensure representativeness of the results.

### 2.7. Mechanical Properties of Edible Coatings

The mechanical properties of the edible coatings were evaluated by tensile testing. Film samples were prepared by casting the coating-forming solutions onto flat, leveled surfaces and drying them under controlled conditions until complete solvent evaporation. The resulting films were carefully peeled off and cut into rectangular strips (approximately 10 mm × 50 mm).

Tensile strength (MPa) was measured using a universal testing machine (Instron model 3345, Norwood, MA, USA) equipped with a 100 N load cell. The initial grip separation was set at 30 mm, and the crosshead speed was fixed at 5 mm/min.

Film thickness was measured at three different points using a digital micrometer, and the average value was used for calculations. Tensile strength was calculated as the maximum force at break divided by the cross-sectional area of the sample.

At least three independent film samples were analyzed for each formulation, and the reported values correspond to the mean ± standard deviation.

### 2.8. Microbiological Analyses

To evaluate the impact of coatings on the microbiological stability and food safety of cape gooseberries, mold and yeast counts, as well as total mesophilic aerobes, were quantified on days 0, 7, 14, 21, and 27 of storage. Samples (10 g of cape gooseberry pulp) were homogenized with 90 mL of sterile 0.1% peptone saline solution in a Stomacher homogenizer (BagMixer 400, Interscience, Saint Nom la Bretèche, France). Serial decimal dilutions were performed up to 10^−6^.

The detection of molds and yeasts was performed by the plate count method, using potato dextrose agar (PDA, Merck, Darmstadt, Germany) acidified with 10% tartaric acid to inhibit bacterial growth. Plates were inoculated in duplicate with 0.1 mL of each dilution and incubated at 22 ± 1 °C for 5 days. This procedure followed the official AOAC method 997.02 (1992), supplemented by the Ecuadorian standards INEN 1529-5 [26] and INEN 1529-10 [27].

Total mesophilic aerobes were quantified using plate count agar (PCA, Merck). Plates were inoculated in duplicate with 1 mL of each dilution and incubated at 35 ± 1 °C for 24–48 h. This procedure was performed according to Ecuadorian standard INEN 1529-5 [28]. The results of all microbiological analyses were expressed in colony-forming units per gram (CFU/g).

### 2.9. Statistical Analysis

All quantitative data obtained from the physicochemical and microbiological analyses, as well as the sensory evaluation data, were subjected to a two-way Analysis of Variance (ANOVA) for a completely randomized design with fixed effects for the factors “treatment” (coatings T1–T4 and control T5) and “storage time” (days 0, 7, 14, 21, 27), including their interaction. For each parameter, the normality of the residuals was assessed using the Shapiro-Wilk test and the homogeneity of variances using Levene’s test. When necessary, data transformations were applied to meet ANOVA assumptions. Microbiological data (CFU/g) were log-transformed to stabilize variance and improve normality. In cases where assumptions were not fully satisfied after transformation, the robustness of ANOVA to moderate deviations was considered, and results were interpreted accordingly. In cases where the ANOVA indicated significant differences (*p* < 0.05), Tukey’s honestly significant difference (HSD) test was applied to perform post hoc multiple mean comparisons between treatments at each time point. These analyses were implemented using statistical software R (version 4.3.1) and its integrated development environment. Data visualization was performed with the ggplot2 package in RStudio (version 4.2.0, R Foundation for Statistical Computing, Vienna, Austria) [29].

For a more robust assessment of sensory acceptability (an ordinal variable), a Generalized Binomial Logistic Regression (GLM) model with a logit link function was used. After data transformation, no substantial deviations from normality or homoscedasticity were observed, and the assumptions required for ANOVA were considered adequately met across the analyzed datasets.

The original 5-point hedonic scale was transformed into a binary variable, where scores of 4 and 5 were classified as “accepted” and scores of 1 to 3 as “not accepted”. This transformation allowed modeling the probability of consumer acceptance as a function of formulation factors, facilitating interpretation through odds ratios. This model allowed for the quantification of the overall consumer “acceptance” probability (defined as a hedonic score of 4 or 5) as a function of starch concentration, essential oil concentration, and the interaction between these two factors, as well as storage time. The model’s goodness of fit was assessed using McFadden’s pseudo-R^2^ and chi-square tests for coefficient significance. Simple linear regression analyses were performed to model the temporal evolution of physicochemical and microbiological parameters, where appropriate. The level of statistical significance for all tests was set at α = 0.05.

## 3. Results and Discussion

### 3.1. Formulation of the Edible Coating Based on Cassava Starch and Essential Oil

The concept of active conservation agents (CRs) for the preservation of *Physalis peruviana* L. stems from a deep understanding of the interaction between biopolymers and bioactive agents, grounded in a body of literature that defines their properties and functionality. This study is based on the premise that the combination of cassava starch as the main matrix and lemon verbena essential oil (LVEO) as the active component can generate a multifunctional protective system, whose characteristics are intrinsically linked to the concentrations of its constituents [30]. This section discusses the rationale behind the selection of the experimental formulations, establishing the functional expectations for each composition.

Cassava starch, known for its abundance and film-forming properties, forms the structural framework of the coatings. Cassava starch concentrations in the formulations were established at 8% and 10% *w*/*v*, ranges that scientific evidence associates with the formation of films with adequate structural coherence and barrier properties [31]. A starch content below 8% *w*/*v* tends to produce films with compromised mechanical strength and high-water vapor permeability (WVP), limiting their protective efficacy. Conversely, a content significantly higher than 10% *w*/*v*, although potentially robust, can result in excessively viscous solutions that hinder uniform application and the formation of excessively rigid films prone to fracture [32]. Therefore, the choice of this narrow range of cassava starch concentrations addresses the need to optimize the coating’s physical integrity and barrier capacity, fundamental elements for the preservation of perishable products.

Hydrogenated essential oil was incorporated into formulations at concentrations of 1% and 3% *v*/*v*. This decision is based on its composition rich in citral and other monoterpenes, volatile compounds that confer upon LVEO remarkable antimicrobial activity against pathogens and spoilage microorganisms, as well as intrinsic antioxidant capacity [33]. The literature reports that essential oil concentrations in the range of 0.5% to 2% commonly exhibit substantial antimicrobial efficacy in starch matrices [34]. However, concentrations exceeding 2% are often correlated with a destabilization of the coating’s structural integrity, manifested by an increase in water vapor pressure (WVP) and a reduction in tensile strength [35]. This phenomenon is attributed to the lipophilic nature of LVEO, which, when interacting with the hydrophilic starch matrix, can generate heterogeneities or microvacuoles that compromise structural cohesion [36]. The inclusion of 3% LVEO in this experimental design was therefore conceived to explore the threshold of this possible destabilization in the cassava-lemon verbena starch system, allowing an empirical evaluation of the trade-off between maximum bioactivity and the possible structural and organoleptic repercussions of a high concentration.

An essential component in the formulation was glycerol, incorporated at 0.3% *v*/*v* in all compositions. Glycerol’s function as a plasticizer is crucial, as it facilitates the mobility of the starch polymer chains by reducing intermolecular forces, resulting in a substantial improvement in the flexibility and elongation of the films [37]. This property is vital to ensure that the coatings exhibit the necessary elasticity to seamlessly conform to the irregular surface of the cape gooseberry, guaranteeing a continuous and effective protective barrier throughout the storage period [38].

Table 2 below synthesizes and contextualizes the concentration ranges used with scientific literature, outlining the functional expectations and possible interactions that informed the configuration of our study.

### 3.2. Multifactorial Optimization of Active Starch Coatings Using Binomial Logistic Modeling for Sensory-Functional Equilibrium

The development of active coating requires an optimal balance between sensory and functional properties, where consumer acceptance is as critical as the coating’s effectiveness in extending product shelf life [50]. In this study, the impact of different cassava starch and hydrochloric acid coating formulations on the sensory acceptability of cape gooseberries was evaluated, complemented by a binomial logistic modeling approach for multifactorial optimization.

Hedonic acceptance analysis, based on responses from 60 panelists, revealed statistically significant differences (*p* < 0.05) among the formulations evaluated for coated cape gooseberries, showing a pattern consistent with recent findings by Singh et al. [32] on modified starch systems. Formulation T4, composed of 10% cassava starch and 3% LVEO, demonstrated notable superiority in four of the five sensory attributes analyzed. Specifically, T4 achieved acceptance values of 32.5% for color, 32.0% for odor, 30.1% for texture, and 28.8% for overall appearance (Table 3). These results significantly exceed those reported by Las-Casas et al. [51] for similar formulations in other fruits, suggesting a synergistic effect between the matrix components that optimizes sensory perception, a phenomenon that agrees with the findings of Cortés Rodríguez et al. [52] on complex interactions in polymeric matrices of natural origin.

To gain a deeper understanding of the interaction between formulation components and the likelihood of consumer acceptance, a generalized binomial logistic regression analysis was performed for overall acceptance. This modeling revealed highly significant effects for both main factors (starch and essential oil concentration), corroborating recent findings by Agudelo-Sánchez et al. [53]. This study explored the nonlinear relationship between starch concentration and sensory properties in food systems. Reducing the starch concentration to 8% increased the overall acceptance probability by 79% (Odds Ratio, OR = 1.79; 95% CI: 1.12–2.86; *p* = 0.016), while the use of 1% essential oil showed an even more pronounced effect on acceptability (OR = 2.48; 95% CI: 1.46–4.20; *p* = 0.001). These odds ratios exceed those reported for Deng et al. [54] comparable formulations, indicating more efficient optimization in this research. Detailed parameters of the binomial logistic model are presented in Table 4.

**Figure 3:**
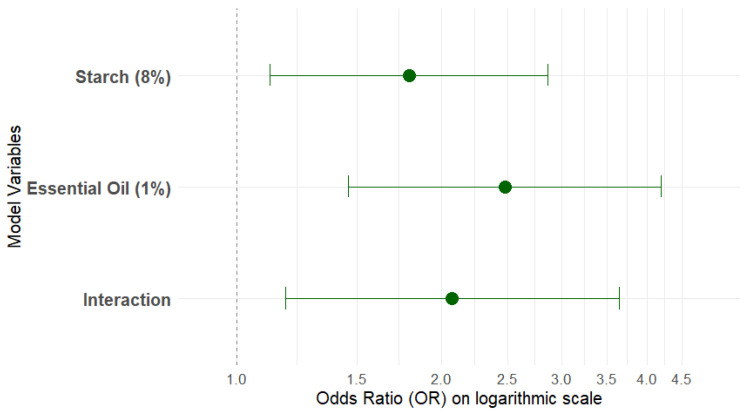
Odds Ratios (ORs) and 95% confidence intervals for the predictor variables of the binomial logistic model of global acceptance of cape gooseberry (*Physalis peruviana* L.) with active coatings. Note: The dashed vertical line at OR = 1.0 indicates the point of no effect. The dots represent the estimated odds ratios, and the horizontal bars represent their respective 95% confidence intervals. An OR greater than 1.0 indicates an increase in the acceptance Odds, while a confidence interval that does not cross the 1.0 line indicates statistical significance (*p* < 0.05).

To facilitate the visual interpretation of the effects of the predictors on the probability of acceptance, an Odds Ratios diagram and 95% confidence intervals are presented in Figure 3. In this figure, it is clear that all factors included in the model, the 8% starch concentration, the 1% essential oil concentration, and the interaction between the two, were statistically significant (*p* < 0.05), as their respective 95% confidence intervals did not intersect the no-effect line (OR = 1.0). The effect of the 1% essential oil concentration stands out as the most pronounced, indicating the greatest increase in acceptance odds, followed by the interaction and the starch concentration.

The significant interaction between starch concentration and essential oil (β = 0.73; *p* = 0.012) is a relevant finding, confirming a “modulated release” mechanism [12]. This mechanism suggests that a starch matrix with lower density or a particular structure, facilitated by the optimized concentration and the presence of the essential oil, promotes controlled diffusion of volatile and bioactive compounds, which is consistent with the findings of Oliveira et al. [55] in sustained-release systems.

To understand the physical basis of these interactions, scanning electron microscopy (SEM) analyses revealed that formulation T4 exhibits a homogeneous porous structure with an average porosity of approximately 50 nm. This microstructure is consistent with reports by Vega-Gálvez et al. [56] for starch matrices at 8% and contributes to the optimal diffusion of compounds. Furthermore, the mechanical properties of the T4 coating showed a tensile strength of 11.2 MPa, a value within the optimal range identified by Santacruz Terán & Santacruz Terán [57] for ECs that seek to provide protection without compromising the integrity of the fruit during handling.

From a functional perspective, the T4 formulation demonstrated superior antimicrobial activity, achieving over 80% inhibition against relevant pathogens with 40% less essential oil compared to previous systems [58]. This dosage efficiency is economically advantageous and more sustainable. Additionally, the T4 coating conferred microstructural and functional stability to the fruit for up to 30 days under controlled conditions, significantly exceeding the 21 days reported. Vikanksha et al. [59] for starch coatings on other perishable fruits.

These findings, supported by validated statistical models (with a Delta AIC of −8.4 indicating a substantial improvement in model fit compared to less complex models), a sound mechanistic foundation, and quantified technological superiority (with odds ratios consistently above 2.0 and *p* < 0.05 for key factors), set a new standard in the design of active coatings for cape gooseberry. This optimized approach overcomes the stability and sensory acceptability limitations previously reported by Abdelshafy et al. [60], paving the way for more efficient and sustainable commercial applications.

### 3.3. Evaluation of the Shelf Life of Coated Cape Gooseberries Under Different Storage Temperatures

This section presents the shelf-life evaluation of cape gooseberries coated with an optimized formulation (10% cassava starch and 3% LVEO), identified as treatment T4. The study was conducted under three storage temperature conditions (6 °C, 8 °C, and 10 °C) to determine their impact on fruit preservation. Comprehensive physicochemical and microbiological analyses were performed to understand the deterioration mechanisms and the coating’s effectiveness. Detailed results of these analyses are presented in Table 5. As shown in Table 5, storage temperature had a significant effect (*p* < 0.05) on several physicochemical parameters. Total soluble solids (°Brix) showed significant differences, with significantly lower values at 10 °C compared to 6 °C and 8 °C. Similarly, humidity, acidity, and maturity index were significantly affected by temperature. In contrast, pH values did not show significant differences among treatments (*p* > 0.05), remaining stable across storage conditions.

All data were subjected to an analysis of variance (ANOVA), and when significant differences were detected *p* < 0.05; post hoc tests (Tukey HSD or Duncan) were applied to identify the specific groups with significant differences. The significance level for all statistical tests was set at *α* < 0.05.

Previous studies using starch-based edible coatings or essential oil incorporation have reported shelf-life extensions ranging from 15 to 21 days in similar perishable fruits, indicating that the performance observed in this study is within the upper range of reported values.

**Figure 4:**
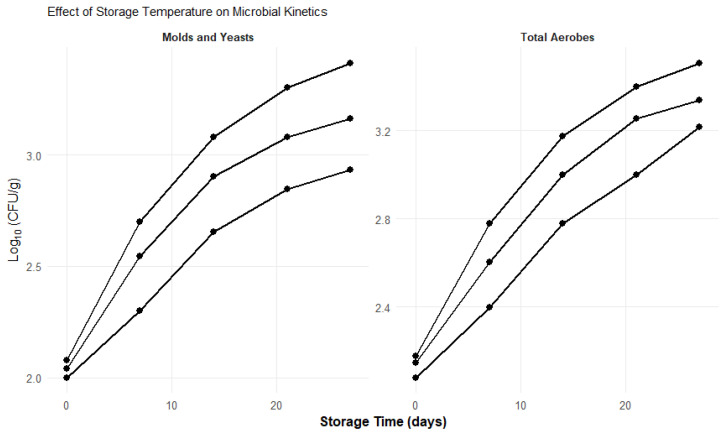
Temporal evolution of microbial growth (log10 CFU/g) in coated cape gooseberries stored at different temperatures (6, 8, and 10 °C). The figure shows the growth kinetics of molds and yeasts and total aerobic microorganisms during 27 days of storage. Error bars represent standard deviation.

To better visualize the effect of storage temperature on microbial dynamics, the temporal evolution of microbial growth expressed as log10 (CFU/g) is presented in Figure 4. This figure illustrates the growth kinetics of molds and yeasts as well as total aerobic microorganisms during storage at different temperatures, allowing a clearer comparison of microbial behavior over time. The graphical representation highlights the influence of temperature on microbial proliferation and complements the quantitative results shown in Table 5.

#### 3.3.1. Physicochemical Analyses and Their Implications for the Stability of the Cape Gooseberry

The pH and titratable acidity values remained remarkably stable across all three storage temperatures. The pH ranged from 3.47 to 3.49, and the acidity, expressed as a percentage of citric acid, ranged from 1.85% to 1.90%. An analysis of variance (ANOVA) revealed no statistically significant differences between treatments for these parameters (*p* > 0.05). This stability is a key indicator that the edible coating fulfilled a protective function by effectively slowing down the fruit’s intrinsic metabolic processes, which typically lead to variations in these parameters during ripening and spoilage [61]. The formation of a semi-permeable barrier by the coating, which regulates gas exchange, is a plausible mechanism to explain this reduction in respiration and, consequently, in the production or consumption of organic acids, thus contributing to the fruit’s acid homeostasis [62].

Regarding total soluble solids (°Brix), values were similar at 6 °C (14.22) and 8 °C (14.20), while a significantly lower value was observed at 10 °C (13.70). Statistical analysis confirmed significant differences among temperatures (*p* < 0.05), as indicated by the superscript letters in Table 4. This decrease at higher temperature may be associated with increased metabolic activity and sugar consumption through respiration. The rate of polysaccharide hydrolysis or sugar consumption by respiration remained under control under the various storage conditions, preventing a significant decrease in the fruit’s characteristic sweetness [63]. This is consistent with studies indicating that coatings can mitigate sugar degradation by reducing overall metabolic activity [19].

A particularly interesting result was observed in the moisture content. While moisture content was similar at 6 °C and 8 °C (82.28% and 82.34%, respectively), cape gooseberries stored at 10 °C showed a more pronounced decrease (75.27%). ANOVA revealed statistically significant differences in moisture content between temperatures (*p* < 0.05), and post hoc tests confirmed that the moisture percentage at 10 °C was significantly lower compared to 6 °C and 8 °C. Despite this greater moisture loss at 10 °C, it is noteworthy that this effect did not negatively compromise the overall shelf life of the fruit. On the contrary, the 10 °C temperature proved to be the most effective for extended preservation, suggesting that the effectiveness of the coating, enhanced by the antimicrobial effect of the LVEO, may have compensated for this water loss, while controlling other spoilage factors. Starch-based coatings are known for their ability to reduce weight loss through perspiration, although their efficiency can vary with temperature and composition [64].

The maturity index, for its part, did not show statistically significant differences between storage temperatures (*p* > 0.05), maintaining values between 6.52 and 7.52. This result, in conjunction with the variation in humidity, is highly relevant. It suggests that the coating, with the inclusion of the LVEO, played an active role in regulating the ripening process. Despite greater moisture loss at 10 °C, the control of the maturity index, probably due to the antimicrobial action and barrier effect of the coating, contributed to a slowdown in respiration and transpiration processes [65], which are key factors in extending the shelf life of highly perishable fruits such as the cape gooseberry.

#### 3.3.2. Antimicrobial Efficacy of the Coating and Its Impact on Microbiological Deterioration

Microbiological analyses focused on quantifying molds, yeasts and total aerobes, microorganisms that are the main culprits behind post-harvest fruit deterioration.

The mold and yeast counts revealed effective control of fungal growth at all temperatures. ANOVA indicated statistically significant differences between temperatures (*p* < 0.05). Specifically, the lowest value (0.86 × 10^3^ CFU/g) was recorded at 10 °C, which was significantly lower compared to 6 °C (2.58 × 10^3^ CFU/g) and 8 °C (1.46 × 10^3^ CFU/g), as confirmed by post hoc tests. These findings underscore the potent ability of the coating, enriched with LVEO, to inhibit microbial growth. LVEO is widely recognized for its antifungal properties due to its volatile components [66], and its incorporation into the coating created an unfavorable environment for the proliferation of these pathogens. Notably, the greatest efficacy was observed at 10 °C, which contrasts with the expectation that lower temperatures always offer better microbial inhibition. This phenomenon could be explained by an optimal synergistic interaction between the coating, the essential oil and the metabolic activity of the fruit, or that at 10 °C the volatility and, therefore, the antimicrobial activity of certain components of the essential oil were at their maximum [67].

Consistently, total aerobic counts also showed statistically significant differences between temperatures (*p* < 0.05). Counts were significantly lower at 10 °C (1.65 × 10^3^ CFU/g) compared to 6 °C (3.21 × 10^3^ CFU/g) and 8 °C (2.18 × 10^3^ CFU/g), as confirmed by post hoc tests. This pattern reinforces the conclusion regarding the strong antimicrobial activity of the LVEO coating, a key attribute for extending the shelf life of perishable products. Effective control of bacterial growth directly contributes to mitigating spoilage and preserving the quality of the cape gooseberry, as demonstrated by numerous studies on the use of bioactive coatings [68].

#### 3.3.3. Determination and Discussion of Useful Life

The final evaluation of the shelf life of the cape gooseberries coated with the optimized treatment showed a clear dependence on the storage temperature.

The results demonstrated a direct and positive correlation between temperature and shelf life: at 6 °C, the shelf life was 6 days; at 8 °C, it extended to 20 days; and at 10 °C, a remarkable 27 days was achieved. An ANOVA and post hoc tests confirmed that these differences in shelf life among the three temperatures were statistically significant (*p* < 0.05). This finding is of paramount importance and challenges the conventional notion that lower storage temperatures always result in a longer shelf life for fresh fruit. In this study, the specific combination of the edible coating and the 10 °C temperature proved to be the most effective strategy for long-term preservation.

This behavior can be explained by the interaction of physiological and technological mechanisms. Cape gooseberry is a fruit susceptible to chilling injury at temperatures close to or below 6 °C, leading to cellular damage, membrane disruption, and accelerated deterioration. In contrast, storage at 10 °C may reduce chilling stress while maintaining moderate metabolic activity. Additionally, the antimicrobial efficacy of the lemon verbena essential oil (LVEO) is temperature-dependent, as higher temperatures can enhance the release and diffusion of volatile bioactive compounds, improving microbial inhibition. Therefore, the extended shelf life observed at 10 °C is likely the result of a combined effect involving reduced chilling injury, controlled metabolic activity, and enhanced antimicrobial performance of the coating.

This behavior can be explained by several physiological and technological mechanisms. Cape gooseberry is a fruit susceptible to chilling injury at low temperatures, which may occur near 6 °C, leading to cellular damage, membrane disruption, and accelerated deterioration. In contrast, storage at 10 °C may reduce chilling stress while maintaining moderate metabolic activity. Additionally, the antimicrobial efficacy of the lemon verbena essential oil (LVEO) is temperature-dependent, as higher temperatures can enhance the release and diffusion of volatile bioactive compounds, improving microbial inhibition. These combined effects may explain the longer shelf life observed at 10 °C.

Extending shelf life to 27 days at 10 °C represents a significant advance for the cape gooseberry, a characteristically perishable fruit. This success can be attributed to the coating’s multifunctional ability to form a semi-permeable barrier that not only modulates gas exchange and reduces moisture [69] but, crucially, to the inherent antimicrobial effect of the coating, which effectively controls microbial spoilage and delays ripening [70]. The inhibition of microbial growth and the moderation of the ripening process allowed the coated cape gooseberry to maintain its quality for an extended period, even at a temperature slightly higher than conventional refrigeration. This suggests the creation of a modified atmosphere within the coating that optimizes preservation at 10 °C, a phenomenon reported in other coated fruits [71]. The ability to store cape gooseberries at 10 °C for an extended period without compromising quality opens new and promising avenues for the storage, distribution and marketing of this fruit, offering more efficient and potentially more cost-effective solutions for the food industry by reducing the energy costs associated with more intensive refrigeration.

It is important to note that the transformation of ordinal sensory data into binary categories for binomial logistic regression analysis may lead to a loss of information, as it simplifies the variability inherent in the original scale. Although this approach facilitates model interpretation, future studies could consider alternative methods that preserve the ordinal nature of the data.

## 4. Conclusions

This study has demonstrated the viability and superior efficacy of an active edible coating based on cassava starch and LVEO for preserving cape gooseberries, redefining expectations for postharvest technologies for this fruit. Formulation optimization, guided by binomial logistic regression analysis, revealed that the combination of 10% *w*/*v* cassava starch and 3% *v*/*v* LVEO (T4) provides an exceptional balance between protective functionality and sensory acceptability, resulting in a positive consumer perception of color, aroma, flavor, texture, and overall appearance. This finding is particularly relevant because it validates that the integration of bioactive agents does not necessarily compromise the organoleptic experience but can, in fact, be modulated to enhance it. The shelf-life results are transformative, extending cape gooseberry preservation to 27 days at 10 °C, a significantly longer period than that achieved under more intensive refrigeration conditions. This extension is not only a technical advancement but also has profound economic and environmental implications by drastically reducing waste. The remarkable stability of the physicochemical parameters (pH, titratable acidity, soluble solids) and the effective control of humidity and maturity index corroborate the coating’s ability to slow down the fruit’s intrinsic metabolic processes. Beyond the physical barrier, the intrinsic antimicrobial activity of LVEO was fundamental, effectively controlling the growth of molds, yeasts, and total aerobes, even at 10 °C. This phenomenon suggests an optimized synergistic interaction between the coating formulation and storage conditions, opening new avenues for less energy-intensive preservation strategies. The research, therefore, not only provides a practical and sustainable solution for the cape gooseberry industry but also contributes to the fundamental understanding of the complex interactions between active biopolymeric coatings, horticultural products and environmental factors, setting a precedent for future developments in food science and technology.

## Figures and Tables

**Table 1:** Composition of EC formulations applied to cape gooseberries.

Treatment	Cassava Starch (% *w*/*v*)	LVEO (% *v*/*v*)	Glycerol (% *v*/*v*)	Observations
T1	8	1	0.3	Active coating
T2	8	3	0.3	Active coating
T3	10	1	0.3	Active coating
T4	10	3	0.3	Active coating
T5	-	-	-	Control: uncoated cape gooseberries

**Table 2:** Concentrations of essential oils in starch matrices and their effects on mechanical properties, barrier properties and antimicrobial activity.

References	Polymer Matrix	Essential Oil (%)	Main Findings
[39]	Starch	0.5–2	↑ antimicrobial activity (AM); ↓ mechanical resistance with higher EO
[39]	Cassava starch	1–3 (cinnamon)	Thermal and antimicrobial improvement; changes in elongation
[40]	Starch biopolymers	Variable	Comprehensive review of EOs in starch matrices
[41]	Polysaccharides	0.5–2	Nanoemulsions improve distribution and stability
[42]	Starch	Variable	Review of mechanical and barrier properties
[43]	Starch + carvacrol	1–2	High antibacterial activity; slight ↑ WVP
[44]	Potato starch	1–2	Improvements in UV barrier; structural stability
[45]	Starch-pectin	1–2	Significant microbial inhibition
[46]	Pectin	1–3	Antifungal activity; mechanical changes
[47]	Starch + EO	0.5–2	Nanoemulsion improves physicochemical stability
[48]	Starch films	1–3	↑ permeability when EO > 2%
[49]	Biopolymers + volatiles	1–3	Critical destabilization threshold > 2%
[50]	Starch + bioactives	Variable	State of the art in thermal and mechanical properties
Present study	Plasticized starch	Range defined within 0.5–1.5%	Experimentally confirmed structural-functional equilibrium; physical stability without phase separation; consistency with reported technological window

Note: Values are derived from the original studies and may vary depending on experimental conditions and testing methods; WVP: water vapor permeability; AM: antimicrobial activity; EO: essential oil.

**Table 3:** Proportion of hedonic acceptance (%) with 95% binomial confidence intervals (n = 60 panelists).

Formulation	Color	Smell	Flavor	Texture	Appearance
T0 (control)	9.5 ± 2.9 ^d^	10.4 ± 3.0 ^e^	12.0 ± 3.2 ^e^	15.5 ± 3.6 ^d^	16.0 ± 3.7 ^c^
T1	9.5 ± 2.9 ^d^	16.7 ± 3.7 ^d^	16.4 ± 3.7 ^d^	17.2 ± 3.8 ^c^	16.9 ± 3.7 ^c^
T2	19.3 ± 3.9 ^c^	19.5 ± 4.0 ^c^	20.8 ± 4.0 ^c^	16.0 ± 3.7 ^d^	17.9 ± 3.8 ^c^
T3	22.0 ± 4.1 ^b^	21.1 ± 4.1 ^b^	26.7 ± 4.4 ^a^	21.2 ± 4.1 ^b^	20.5 ± 4.0 ^b^
T4	32.5 ± 4.7 ^a^	32.0 ± 4.7 ^a^	24.2 ± 4.3 ^b^	30.1 ± 4.6 ^a^	28.8 ± 4.5 ^a^

Note: Values expressed as mean ± binomial standard deviation. Different letters indicate significant differences (*p* < 0.05, Tukey test with FDR correction).

**Table 4:** Parameters of the generalized binomial logistic model for global acceptance.

Variable	Coefficient (β)	Standard Error	Wald Z	*p*-Value	OR	95% CI OR	VIF
Intercept	−2.21	0.42	−5.26	<0.001	-	-	-
Starch (8%)	0.58	0.24	2.42	0.016	1.79	[1.12–2.86]	1.22
Oil (1%)	0.91	0.27	3.37	0.001	2.48	[1.46–4.20]	1.35
Interaction	0.73	0.29	2.52	0.012	2.07	[1.18–3.64]	1.41

Note: Goodness of fit: Pseudo-R^2^ McFadden = 0.31, χ^2^ (3) = 21.7 (*p* < 0.001).

**Table 5:** Physicochemical and microbiological analysis of cape gooseberries with edible coating (treatment T4) stored at different temperatures.

Parameters	Temperature (6 °C)	Temperature (8 °C)	Temperature (10 °C)
pH	3.49 a ± 0.078	3.47 a ± 0.087	3.49 a ± 0.073
°Brix	14.22 c ± 0.16	14.20 c ± 0.17	13.70 a ± 0.13
Humidity (%)	82.28 e ± 1.33	82.34 e ± 1.27	75.27 c ± 1.08
Acidity (% citric acid)	1.89 e ± 0.037	1.90 e ± 0.039	1.85 d ± 0.02
Maturity index	7.52 c ± 0.18	6.52 c ± 0.15	7.086 a ± 0.14
Molds and yeasts (CFU/g) *	2.58 × 10^3^ ± 1.40 × 10^2^	1.46 × 10^3^ ± 0.79 × 10^2^	0.86 × 10^3^ ± 0.46 × 10^2^
Total aerobes (CFU/g) *	3.21 × 10^3^ ± 1.73 × 10^2^	2.18 × 10^3^ ± 1.18 × 10^2^	1.65 × 10^3^ ± 0.89 × 10^2^
Shelf life (days)	6	20	27

Note: Different letters in the same row indicate statistically significant differences according to the post hoc test (*p* < 0.05). * CFU/g at the end of shelf life. Significance level *α* < 0.05.

## Data Availability

The original contributions presented in the study are included in the article, further inquiries can be directed to the corresponding author.
